# Adopting and implementing an innovative model to organize diabetes care within First Nations communities: A qualitative assessment

**DOI:** 10.1186/s12913-021-06424-1

**Published:** 2021-05-03

**Authors:** Lisa A. Wozniak, Allison L. Soprovich, Jeffrey A. Johnson, Dean T. Eurich

**Affiliations:** Alliance for Canadian Health Outcomes Research in Diabetes, School of Public Health, 2-040 Li Ka Shing Centre for Health Research Innovation, University of Alberta, Edmonton, Alberta T6G 2E1 Canada

**Keywords:** First Nations, Type 2 diabetes, Health services delivery, Qualitative assessment, Adoption, Implementation

## Abstract

**Background:**

Diabetes care remains suboptimal in First Nations populations. Innovative and culturally relevant approaches are needed to promote systematic and proactive organization of diabetes care for people living with diabetes on-reserve in Canada. The RADAR model is one strategy to improve care: an integrated disease registry paired with an electronic health record for local community healthcare providers with remote care coordination. We qualitatively assessed adoption and implementation of RADAR in First Nations communities in Alberta to inform its potential spread in the province.

**Methods:**

We used the RE-AIM framework to evaluate adoption and implementation of RADAR in 6 First Nations communities. Using purposeful sampling, we recruited local healthcare providers and remote care coordinators involved in delivering RADAR to participate in telephone or in-person interviews at 6- and 24-months post-implementation. Interviews were digitally recorded, transcribed, and verified for accuracy. Data was analyzed using content analysis and managed using ATLAS.ti 8.

**Results:**

In total, we conducted 21 semi-structured interviews (6 at 6-months; 15 at 24-months) with 11 participants. Participants included 3 care coordinators and 8 local healthcare providers, including registered nurses, licensed practical nurses, and registered dietitians. We found that adoption of RADAR was influenced by leadership as well as appropriateness, acceptability, and perceived value of the model. In addition, we found that implementation of RADAR was variable across communities regardless of implementation supports and appropriate community-specific adaptations.

**Conclusions:**

The variable adoption and implementation of RADAR has implications for how likely it will achieve its anticipated outcomes. RADAR is well positioned for spread through continued appropriate community-based adaptations and by expanding the existing implementation supports, including dedicated human resources to support the delivery of RADAR and the provision of levels of RADAR based on existing or developed capacity among local HCPs.

**Trial registration:**

Not applicable to this qualitative assessment. ISRCTN14359671.

**Supplementary Information:**

The online version contains supplementary material available at 10.1186/s12913-021-06424-1.

## Background

Diabetes rates among Indigenous peoples globally is disproportionate [1]. In Canada, the prevalence of diabetes is 3–5 times higher [2] and mortality rates 2–3 times higher for First Nations people than the general population [3]. These issues are compounded by suboptimal diabetes care, particularly in rural or remote settings where many First Nations people live [2, 4–6]. In Canada, diabetes care in First Nations communities is delivered through federally-funded nurse-led homecare, community health, and diabetes programs with limited access to primary care physicians and specialists [7] Additionally, healthcare providers in some First Nations communities in Alberta feel limited in their ability to identify, track, and manage patients with type 2 diabetes; therefore, care was typically reactive and dependent on patients’ abilities to navigate the health system [8]. Clearly, there is a need for innovative and culturally-relevant approaches to promote systematic and proactive organization of diabetes care for First Nations people living with diabetes on-reserve in Canada [9, 10].

**Fig. 1:**
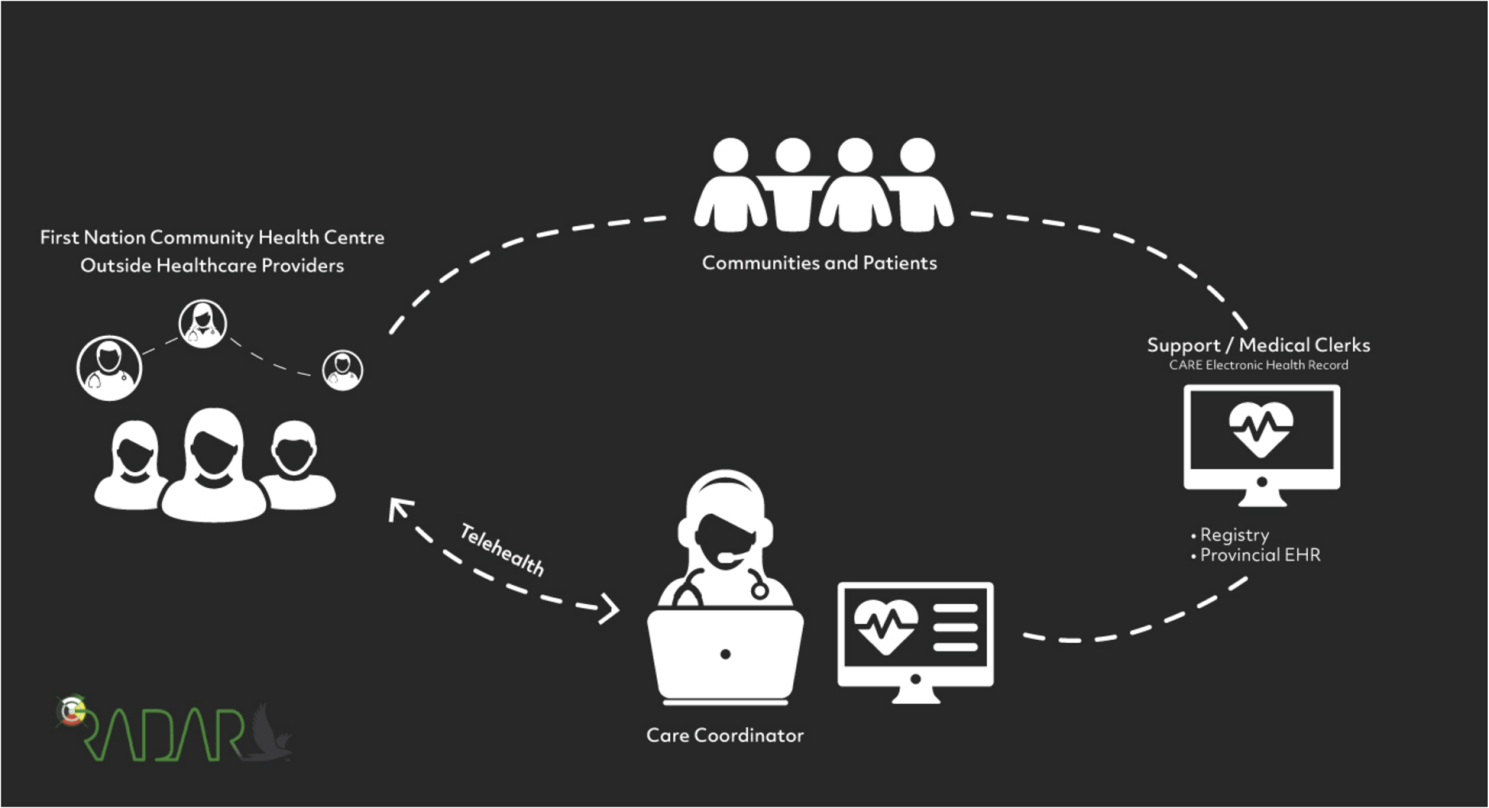
RADAR Infographic

In response to this need, the RADAR model was developed by First Nations communities and OKAKI Health Intelligence Inc. (hereafter **OKAKI**), a private sector social-enterprise, company in Alberta, Canada, with > 20 years working with First Nation communities. All communities had representation on the steering committee to guide the project. **RADAR** stands for **R**eorganizing the **A**pproach to **D**iabetes care through the **A**pplication of **R**egistries and is described in detail elsewhere [11]. Briefly, RADAR consists of local healthcare providers in First Nations communities supported by remote care coordinators, who are registered nurses, through telehealth representing the care team (Fig. 1).

Within this remote-support model, the team work collaboratively to:

1. Use a shared electronic health record/diabetes registry called **CARE**, containing clinical patient data on key diabetes outcomes and quality of care indicators populated and maintained by local health care providers and remote care coordinators; and,
2. Coordinate population-level care to identify gaps, recommend and/or implement therapeutic changes, and coordinate referrals using current clinical practice guidelines through regular case review and conferencing.

RADAR is being implemented collaboratively with several First Nations communities from Treaty 6, 7, and 8 territories in Alberta, Canada. Several steering committee meetings with community representation were held prior to implementation to adapt the RADAR model to meet local needs to promote success of the program within each community. Prior to RADAR, the participating health centres had already implemented CARE for their home care programs; however, CARE was not being used specifically for diabetes care at this point.

Regardless of the promise of this collaborative and innovative model, there are considerable challenges to translating interventions into practice [12, 13] and research on the adoption and implementation of interventions in a variety of practice settings is needed [14, 15]. Indeed, each First Nation community has unique strengths and challenges to managing health on-reserve. Understanding the implementation of diabetes-related interventions in First Nations communities is particularly important given the tremendous potential to improve diabetes management through service delivery. Therefore, our purpose was to qualitatively evaluate the adoption and implementation of RADAR in health centres in six First Nations communities to inform its potential spread.

## Methods

This qualitative assessment was part of a prospective controlled trial evaluating RADAR’s effectiveness [11] and allowed us to elicit experiences from individuals directly involved in delivering RADAR [16]. We used the RE-AIM framework, which has been used to evaluate the impact of public health interventions, including diabetes self-management interventions, to support decision-making [17–24]. RE-AIM consists of five dimensions related to the processes and outcomes of interventions: Reach into the target population; Effectiveness of the intervention; Adoption by targeted end-users; Implementation, including consistency; and Maintenance of intervention effects over time. This evaluation focused on the adoption and implementation (i.e. processes) of RADAR by First Nations communities.

### Data collection

RADAR is expanding to additional First Nations communities in Alberta, with communities added sequentially in consecutive 4–6 month periods based on community readiness. Our qualitative assessment was based on the first 6 communities to adopt and implement RADAR, which were diverse by treaty, geography, population, and proximity to urban centres, offering a range of experiences. We used purposeful sampling of participants directly involved with RADAR. Community health managers identified potential participants who were sent an introductory email outlining the purpose of the evaluation and an invitation to participate in telephone or in-person interviews. Interested participants contacted the research team by telephone or email to learn more about the evaluation and arrange participation. We recruited remote care coordinators (**CCs**) at 6- and 24-months post-implementation. We recruited local healthcare providers (**HCPs**) at 24-months post-implementation only to reduce participant burden, a strategy fully endorsed by community health managers, and to enable them to become familiar with RADAR to comment on its adoption and implementation. Multiple attempts were made to recruit participants involved in the implementation of RADAR at the 6 participating health centers for the purposes of data saturation. A trained qualitative researcher (LAW) with no prior relationships with participants conducted the interviews using semi-structured guides, which were refined as data analysis progressed (available upon request). Interviews ranged from 60 to 150 min and were digitally recorded, transcribed, and verified for accuracy.

First Nations leaders (e.g., Chiefs and Council, and community health managers) in the participating communities reviewed and endorsed the project. Formal research and data sharing agreements were completed with each community as outlined in the OCAP principles [25]. Approval for the RADAR project was obtained from the University of Alberta’s Health Research Ethics Board (Study ID Pro00048714). The Health Research Ethics Board determined that the qualitative assessment of RADAR did not require written consent. Regardless, all qualitative methods were carried out in accordance with requirements outlined in the Canadian Tri-Council Policy Statement: Ethical Conduct of Research Involving Humans [26] and all participants provided verbal informed consent.

### Data analysis

Two researchers (LAW and DTE) applied content analysis to the data [27]. First, we coded all data related to adoption and implementation of RADAR. Then, we used an inductive approach to identify recurring codes and concepts across participants and health centre settings. We reviewed code definitions and emerging concepts at regular research team meetings and discussed discrepancies to reach consensus. In addition, findings were presented to community health managers and staff for feedback on the interpretation of data, which included a formal presentation of the findings, as well as review and feedback on the draft and final versions of this manuscript. We conducted concurrent data collection and analysis for the purposes of data saturation [28]. All data was managed using ATLAS.ti Version 8, including documentation of research activities, decisions, and reflections [29]. As described above, we used well-established strategies to ensure rigour, including methodological coherence, collecting and analyzing data concurrently, peer debriefing, maintaining an audit trail using qualitative data analysis software, and reported our findings following the consolidated criteria for reporting qualitative research (COREQ) (Supplemental File 1) [27, 30–32].

## Results

In total, we conducted 21 telephone or in-person interviews with 11 individuals. At 6-months post-implementation, between May 2015 and January 2018, we conducted 6 interviews with 2 care coordinators. At 24-months post-implementation, between April 2016 and February 2019, we conducted 15 interviews with 3 care coordinators and 8 local health care providers, including registered nurses, licensed practical nurses, and registered dietitians (Table 1).

**Table 1:** Interview timing and participant roles

Participant role	Interviewed at 6-month (#)	Interviewed at 24-month (#)	Total (#/%^**a**^)
Care Coordinator	2	3	3 (27%)^**b**^
Dietitian		3	3 (27%)
Licensed Practical Nurse		2	2 (18%)
Registered Nurse		3	3 (27%)

^a^Percentages were rounded and, therefore, may not equal 100

^b^2 care coordinators were interviewed at both 6- and 24-months

We found that the adoption of RADAR was influenced by leadership support as well as perceptions related to the appropriateness, acceptability, and value of the model. In addition, we found that implementation of RADAR was variable across communities regardless of implementation supports and appropriate community-specific adaptations. The 5 themes are described and categorized by adoption or implementation with illustrative quotes with participant role.

### Adoption

The adoption of RADAR was influenced by leadership support as well as participants’ perceptions related to the appropriateness, acceptability, and value of the model.

#### Variable leadership support

The adoption of RADAR was influenced by leaders across levels (i.e., health directors or managers, Tribal Chief and Council, and local HCPs) and their willingness to innovate, provide human resources, and/or champion RADAR. Participants described, *“support from our director in participating”* (HCP) and *“the approval from our chief in council”* (HCP) as influencing factors. Participants also described peoples’ willingness to innovate including *“The health director wants to help her community and make a difference and she is willing and open to make changes”* (CC) and *“We have a team that is open and willing to changing the way things are done to improve”* (HCP). Providing the necessary human resources also facilitated adoption of RADAR, with a few health managers expanding their RADAR teams from 1 local HCP to include additional HCPs for support (CC; HCP). In addition, a care coordinator explained that while leadership support from managers was necessary, local HCPs who were willing and interested in participating in RADAR were essential, and acted as champions: “*If you don’t have a person who wants to do the job, then it’s not about the managers*” (CC). Indeed, some HCPs took initiative to lead RADAR: “*I talked with the care coordinator and our health director and said, ‘I can do this. I am interested in this’”* (HCP). This local HCP modeled the delivery of RADAR for other HCPs and “*demonstrated to the rest of the team how this could work*” (CC). In contrast, minimal leadership support by health directors or managers in some communities was a barrier to adopting RADAR fully, including being too busy “*in their own trenches*” to focus on diabetes-related population trends (CC) or reluctant to provide local HCPs with the tools necessary for RADAR (e.g. provincial EMR access) (CC).

#### Model perceived as appropriate

The adoption of RADAR, including CARE, was influenced by participants’ perception that the model was appropriate to local HCPs and the patient population. RADAR was *“specifically designed for diabetes care”* (CC) and for local HCPs working in First Nations communities. A local HCP described how RADAR “*provides our community with those fundamentals [of diabetes care] that are necessary to provide effective client care and still allows our community freedom to decide how we want to deliver the care*”. Indeed, care coordinators explained that RADAR, including CARE, “*makes sense to [HCPs] and their world*” (CC) and “*works well with the workflow in a clinic*” (CC). Another CC stated *“most EMR electronic health records are physician-based and designed for a doctor to use whereas CARE was designed for a nurse who is actually in the communities providing patient care*”.

However, while participants considered RADAR appropriate overall, it was less appropriate in a community with an existing Certified Diabetes Educator (**CDE**). Based on the skills of the CDE, this community did not need the same level of support from RADAR as others. Therefore, appropriateness of RADAR “*really depends on the community*” and their human resources (HCP). Participants suggested tailoring RADAR to the needs of local HCPs to offer the right level of support, from full to minimal support (CC). Furthermore, RADAR could be adjusted in communities that have participated for several years, where HCPs have gained the confidence and capacity to deliver diabetes care with less support (CC). Regardless, participants commented, *“there’s always going to be a need for RADAR”* in some form to sustain its adoption due to significant staff turnover within communities (CC). As one HCP said, “*I worry once this project is over that our community will not be able to sustain [RADAR] on our own. We have such a high staff-turnover rate and new staff members would have to be oriented to CARE, to diabetes, to their role on the care team. Without the case coordinator acting as that stable source of support, I think the overall goal of RADAR and that big picture is going to get lost*”.

RADAR was also perceived as appropriate to the patient population, predominantly First Nations people. A HCP explained RADAR “*was a good fit for the community, for the diabetes population, and culturally appropriate*”. Participants viewed RADAR, including CARE, as a clinically-based tool and it was *“how you frame it to clients that is your cultural context*” (HCP). A care coordinator agreed it was the responsibility of HCPs to use clinical information from CARE appropriately with patients: “*It is a useful tool, and HCPs dictate if it’s culturally appropriate in their interactions and how respectful they are with the clients*” (CC). Regardless, a care coordinator recommended enhancing RADAR’s appropriateness for the patient population by capturing additional information in CARE within designated fields, rather than narrative charting, including use of traditional medicines (CC) and socioeconomic information like income or food security (CC).

#### Model and technology perceived as acceptable

Overall, participants found RADAR’s remote-support model and accompanying technology, GoToMeeting™, acceptable. A HCP explained, “*We use* GoToMeeting™ *for our case conferences. Everyone sits at their computer and the care coordinator shares her screen with us. Everyone sees the chart notes for that client and any other information in CARE … I like our case conferences, I like using GoToMeeting™. I think it is working really well*”. Another HCP described case conferencing as “*an efficient use of time*”.

Participants also found the electronic health record platform, CARE, acceptable. Facilitators to adopting CARE included previous use in home care programs and its user-friendly nature. HCPs explained, “*It’s natural because we’ve used CARE for quite a while*” and “*I’ve been using CARE to chart for quite a while so I’m confident with it*”. Similarly, a care coordinator commented that some HCPs *“were already using CARE [in home care], so that made training easier*” (CC). Participants also described CARE as “*the most user-friendly EMR that I have used*” (CC), “*pretty straight forward*” (HCP), and easy for “*our less computer savvy or techno-adverse care providers”* (CC). Lastly, OKAKI improved the functionality of CARE based on feedback from HCPs and care coordinators: “*OKAKI is constantly making improvements to CARE and are very open to feedback as to how it could work better, or if we could do something differently that would help us out more*” (HCP). Other health centers might be willing to adopt RADAR because the remote-support model and associated technology (CARE and GoToMeeting™) was acceptable to these 6 communities.

Regardless, it took HCPs and CCs time to adjust to this remote-supported model. Some local HCPs wanted in-person, rather than remote, communications with care coordinators so care coordinators adapted the model through regular site visits: “*[HCPs] want to see someone; they want to chat in person, not over the internet. The first few months they wanted me to come on a monthly basis to do case conferences. I said, ‘That defeats the purpose, but I’ll come every 3 months’. So, it took them awhile to adjust to it*” (CC). A HCP explained the importance of in-person visits to develop relationships: “*It’s nice because the care coordinator comes to the community every once in a while for a face-to-face so you get to know the person you’re talking with through the computer”*. Similar to HCPs, a care coordinator described adjusting to the remote-support model and not providing direct patient care: “*That’s a struggle every day for me because I’m a clinical nurse. But I am adjusting to it … The remote nature, sitting in my office, not actually speaking the clients*. This care coordinator accepted her role upon realizing she leveraged her skills by supporting HCPs to support patients: “*I’m getting used to it … I can train HCPs and make sure they’re asking the right questions*”.

#### Model perceived as valuable

The adoption of RADAR was also influenced by its perceived value to support local HCP practice and address diabetes care gaps. Some local HCPs saw *“the value in RADAR and how it’s making it easier for them to do their job and to provide better care*” (CC) and the potential of RADAR to address their “*need for better diabetic care”* (CC). For example, before RADAR “*we were looking for something other than what we were doing to track our clients because we were finding people were falling through the cracks*” (HCP). Another HCP described the value of accessing care coordinators because the health center received limited resources from the province: “*It works well calling the care coordinator or OKAKI to help us through whatever problem or issue there is, as a resource. Because, you’re not going to get that from Alberta Health Services because they have a different system*”. HCPs who recognized the value of RADAR facilitated its adoption explaining to care coordinators *“We need this program, it’s a good idea”* (CC) or *“We’ll find a way, we’ll make [RADAR] work”* (CC).

However, some local HCPs did not value RADAR in part “*because they didn’t really understand RADAR”* (CC) making adoption difficult. A new HCP to the RADAR team explained, “*nobody’s ever really explained RADAR to me, what to do and what it’s all about*”. In other cases, local HCPs did not value RADAR because they considered it redundant to care they were providing: “*They didn’t want to do this project because in their mind they’re thinking, ‘We’re already doing this*’” (CC). Lastly, in some health centres, healthcare providers not directly involved in RADAR were unaware of it, limiting its full adoption: “*RADAR is not a recognizable program here … Not everybody is aware of what RADAR is. I bet half my co-workers don’t even know what it’s all about. It is a bit of a problem*” (HCP). As such, a care coordinator recommended regular communication with all health center members to increase awareness of RADAR and its value, especially in the context of frequent staff turnover: “*Sometimes the staff change, turnover … So, talking about RADAR again, six months later. And then doing it again six months later*” (CC).

### Implementation

Overall, participants reported sufficient supports to implement RADAR while allowing for community-specific adaptations to the model. Regardless, RADAR was not fully implemented as intended in all communities.

#### Sufficient implementation supports and community-specific adaptations

In general, participants perceived the supports and training provided to implement RADAR sufficient. Participants described multiple implementation supports including a help desk, development of training manuals, and support from CCs. To support implementation, OKAKI provided a “*help desk*” for local HCPs: “*We always have the support there. When we have a question, whether it be with RADAR or CARE, it’s just a phone call away*” (HCP). However, some HCPs sought support from care coordinators instead of the help desk as intended: “*Sometimes [HCPs] call me and ask CARE questions … I don’t mind taking those calls if I have time, but really they should go to help desk*” (CC). In addition, OKAKI developed training manuals for HCPs on how to implement RADAR as intended; however, these manuals were not available for the first communities delivering RADAR.

Support from the care coordinators included demonstrations for the HCPs on how to use CARE, including for patient care, during case conferences: “*[Care coordinator name] shows us a lot through our RADAR meetings, she’ll show us a lot of different ways to access stuff on CARE*” (HCP). However, demonstrations by care coordinators were not helpful for all HCPs: “*She can pull it up for us and do it really fast, but it’s not helping. We need to do it ourselves*” (HCP). Regardless, care coordinators were readily available by telephone or email, *“not just during our case conferences*” (HCP), to support HCP use of CARE because “*you’d forget the first few times how you did that, and we’d email [care coordinators for reminders]*” (HCP). As such, care coordinators recognized the need for “*continually training*” (CC) of HCPs to support the full use of CARE, including “*supplemental education of the health centre staff on the CARE system*” (CC).

The implementation of RADAR allowed for community-specific adaptations because “*there’s a lot of similarities between communities and a lot of differences too*” (HCP). RADAR was adapted because every community was “*unique in their human [and financial] resources to build on the program*” (CC). Care coordinators worked with local HCPs and “*with the skills and whatever they have*” (HCP) to adapt RADAR implementation to be *“individualized for our community*” (HCP).

#### RADAR implementation varied across communities

Regardless of implementation support and community-level adaptations, the implementation of RADAR as intended, including use of CARE and case conferencing, varied by HCPs and/or community. For example, there was limited use of CARE by some HCPs, including “*It’s hard to say how much [HCPs] use [CARE]*” (CC) or *“[HCP] would never log onto CARE when we were case conferencing*” (CC). In addition, some HCPs did not use CARE as intended, such as entering patient data (e.g. “*Some of the medications on my clients, I have not completed*” (HCP)) or using all of its features/tools (e.g. “*Not everyone is tracking the tasks that they have completed with their clients*” (HCP)). Furthermore, some HCPs did not know how to use CARE to inform patient care even when they understood its mechanics: “*They know how to use CARE and it makes sense. They know how to chart, enter meds. They know there’s a health profile they need to update if [patients] have high blood pressure. But I don’t think they knew how they can use it to provide patient care*” (CC).

In addition, case conferences between local HCPs and remote care coordinators varied by community, including the frequency (i.e., weekly, bi-weekly, or monthly) and duration (i.e., 30 to 120 min per case conference), often related to availability: *“[HCPs] don’t have time to [meet weekly], so we meet biweekly or once a month. It really depends on their availability*” (CC). In addition, HCP attendance at case conferences varied by community from challenges to “*having all of our team members show up for our case conferences”* (HCP) to no HCPs attending: “*they just didn’t show up to case conference*” (CC). Often, poor attendance was related to scheduling issues, vacation, or staff turnover: “*There was a lot of case conferences cancelled due to [HCP’s] work schedule”* (CC).

## Discussion

We found variable adoption and implementation of RADAR as intended across 6 First Nations communities. Whether RADAR was adopted fully depended on leadership support and participants’ perceptions of the model’s appropriateness for local HCPs and the patient population, acceptability of the remote-support model and associated technology, and value in supporting local HCP practice to address diabetes care gaps. In addition, RADAR was not fully implemented across some communities despite implementation supports and community-specific adaptations. The partial adoption and implementation of RADAR has implications for how likely it will achieve its anticipated outcomes [33].

Certainly, there was variable readiness across the 6 communities to adopt and implement RADAR. This included limited motivation or tension to change as demonstrated by managers and HCPs being too busy “in the trenches” to focus on diabetes-related population trends. Some local HCPs did not value RADAR because they saw it as redundant to care they were already providing. As such, the readiness of organizations or the fit of RADAR for communities and/or the development of targeted messaging to promote motivation to change among leaders and local HCPs may require assessment [34, 35]. It is critical to co-design appropriate messaging to fully engage and motivate the people and settings who adopt and implement innovated interventions, like RADAR, to optimize uptake [36]. Tools, like Mapping the System, can be used to help understand participants’ values as well as potential losses and benefits as a result of implementing a new model, process, or intervention [35]. In the case of RADAR, targeted messaging for community leaders and health centre staff, might include reframing diabetes care from reactive and episodic to proactive and preventative [8]. Messaging could capitalize on health directors’ and local HCPs’ focus on traditional public health, such as immunizations, to prevent disease and reframe diabetes care similarly as prevention of chronic disease [37].

To increase its potential to achieve outcomes, the spread (i.e. future adoption and implementation) of RADAR could be supported through the use of existing implementation frameworks such as the He Pikinga Waiora Implementation Framework [38], developed for indigenous communities in general, the Consolidated Framework for Implementation Research (CFIR) [39], or Integrated Promoting Action on Research Implementation in Health Services (i-PARIHS) [40]. These frameworks help contextualize implementation across settings to inform why or how interventions were effective or not [41]. Specifically, they can be used to predict, understand, and monitor the interplay of characteristics of interventions, like RADAR, and contextual factors for health centers and communities such as leadership and perceived appropriateness, acceptability, and value of the intervention. Through application of implementation frameworks, the readiness needs and implementation facilitators and barriers can be documented and addressed to promote spread and scale [42].

Indeed, we found each community was unique. RADAR proved adaptive because the model was designed to address the needs of communities in a flexible, rather than prescriptive, manner. Indeed adaptations to models, like RADAR, should be anticipated as they are necessary to successful implementation and, thus, effectiveness [43]. We found that RADAR was adapted based on existing or developed capacity among local HCPs over time. Going forward, the implementation of the RADAR model should consider offering ‘levels of RADAR’ for communities that require less support or reduced care coordinator support because of existing capacity or for “graduates” of the full RADAR model. Regardless, it will be crucial to further document adaptations to RADAR in subsequent communities to understand how modifications influenced its anticipated outcomes [44].

The fact that RADAR was not fully implemented as intended in all communities regardless of implementation supports indicates the need for further supports. The ability to support implementation well requires competencies and time. The Interactive Systems Framework for Dissemination and Implementation outlines distinct roles needed to implement evidence-based innovations to achieve outcomes including [1]: synthesis and translation to distill information for evidence-based intervention [2]; delivery of the intervention; and [3] support to build-capacity among people delivering the intervention [45]. The role of support to build-capacity is the least developed [46]. Indeed, for RADAR, care coordinators performed the roles of delivering RADAR with local HCPs as well as supporting HCPs’ capacity to delivery RADAR. Local HCPs often relied on care coordinators rather than the help desk for support delivering RADAR, including how to use CARE. As such, the RADAR model might consider seconding experienced care coordinators into the role of ‘implementation coach’ to support delivery of RADAR and hire additional care coordinators who are only responsible for delivering RADAR in partnership with local HCPs. Through clarifying roles and responsibilities, organizations like OKAKI, are well-positioned because they have already developed the necessary tools (i.e., CARE), training (i.e., training manuals), technical assistance, and quality assurance (i.e. help desk) needed to bridge interactions between the support and delivery roles [47].

Our results should be interpreted in light of several limitations. While the communities were diverse, our findings were based on the experiences of healthcare providers from 6 voluntary health centres that may not be representative of other health centres on-reserves. As with all qualitative research, there may be limited transferability of the results beyond the present context (i.e. sample and setting). Regardless of its limitations, the strengths of this work include its qualitative descriptive approach and contribution to the current literature by describing the adoption and implementation of an innovated model for diabetes care in First Nations communities in Alberta to inform its potential spread.

## Conclusions

We found variable adoption and implementation of RADAR across 6 communities, which has implications for how likely it will achieve its anticipated outcomes. Regardless, OKAKI is well-positioned to improve the adoption and implementation of RADAR in current and future communities through community-based adaptations and existing implementation supports. The expansion of implementation supports, including dedicated human resources to support the delivery of RADAR by care coordinators and local HCPs and the provision of “levels of RADAR” based on existing or developed capacity among local HCPs, may support its potential spread.

## Supplementary Information

**Additional file 1.**

## Data Availability

The datasets generated and/or analysed during the current study are not publicly available due the potential to identify individuals and/or communities involved, but are available from the corresponding author on reasonable request.
